# Monitoring Migraine Cycle Dynamics with an Easy-to-Use Electrophysiological Marker—A Pilot Study

**DOI:** 10.3390/s18113918

**Published:** 2018-11-14

**Authors:** Goded Shahaf, Pora Kuperman, Yuval Bloch, Shahak Yariv, Yelena Granovsky

**Affiliations:** 1 BrainMARC LTD, P.O Box 128, Yokneam 20692, Israel; 2 The Laboratory of Clinical Neurophysiology, The Rappaport Faculty of Medicine, Technion—Israel Institute of Technology, Haifa 31096, Israel; poraswu@gmail.com (P.K.); y_granovsky@rambam.health.gov.il (Y.G.); 3 The Emotion-Cognition Research Center, Shalvata Mental Health Care Center, Hod-Hasharon 45100, Israel; yuvalbl@clalit.org.il; 4 Sackler Faculty of Medicine, Tel-Aviv University, Tel Aviv 6997801, Israel; 5 Department of Psychiatry, Emek Medical Center, Afula 1834111, Israel; Shahak.Yariv@camh.ca; 6 Department of Neurology, Rambam Medical Center, Haifa 3655306, Israel

**Keywords:** migraine, EEG, migraine phase, attention, brain engagement index

## Abstract

Migraine attacks can cause significant discomfort and reduced functioning for days at a time, including the pre-ictal and post-ictal periods. During the inter-ictsal period, however, migraineurs seem to function normally. It is puzzling, therefore, that event-related potentials of migraine patients often differ in the asymptomatic and inter-ictal period. Part of the electrophysiological dynamics demonstrated in the migraine cycle are attention related. In this pilot study we evaluated an easy-to-use new marker, the Brain Engagement Index (BEI), for attention monitoring during the migraine cycle. We sampled 12 migraine patients for 20 days within one calendar month. Each session consisted of subjects’ reports of stress level and migraine-related symptoms, and a 5 min EEG recording, with a 2-electrode EEG device, during an auditory oddball task. The first minute of the EEG sample was analyzed. Repetitive samples were also obtained from 10 healthy controls. The brain engagement index increased significantly during the pre-ictal (*p* ≈ 0.001) and the ictal (*p* ≈ 0.020) periods compared with the inter-ictal period. No difference was observed between the pre-ictal and ictal periods. Control subjects demonstrated intermediate Brain Engagement Index values, that is, higher than inter-ictal, yet lower than pre-ictal. Our preliminary results demonstrate the potential advantage of the use of a simple EEG system for improved prediction of migraine attacks. Further study is required to evaluate the efficacy of the Brain Engagement Index in monitoring the migraine cycle and the possible effects of interventions.

## 1. Introduction

Migraine is a prevalent disease, affecting more than 15% of females and more than 5% of males [1]. Migraine attacks may cause significant discomfort and reduced functioning for days, including the pre-ictal and post-ictal periods [2]. During inter-ictal periods, however, migraineurs seem to function normally. It is puzzling that synchronized cortical responses to external stimuli or event-related potentials (ERPs) of migraine patients often differ also in the asymptomatic, inter-ictal phase [3]. A frequently reported finding for the differentiation in inter-ictal migraine seems to be reduced habituation. ERP habituation is the degree of reduction in response to stimuli between the initial and subsequently sampled blocks. Such habituation is observed in control patients, but is found to be reduced in migraineurs. Reduced habituation manifests in both early ERP waves, peaking within 200 milliseconds of the stimulus [4,5], and in later ERP waves [6,7]. Early ERP waves, possibly peaking up to 200 milliseconds after the stimulus, are often associated with perception- and sensation-related processes; later ERP waves are often associated with attention-related processes [8]. ERP patterns have been observed to change before a migraine attack. The changes, often reported 12–48 h before an attack, seem to involve an increase in initial response and in habituation (pseudo-normalization), both in early and later ERP waves [7,9,10,11].

The mechanism responsible for reduced habituation during the inter-ictal phase is not clear. It has been suggested that it is an epiphenomenon of reduced response in the initial sample blocks, which sets limits upon the possible range of habituation [12,13,14]. This possibility motivates the attempt to harness an electrophysiological marker for extraction of effective ERP wave-related information from the initial sample blocks. 

Over the last several years we have developed an effective single-electrode marker for attention. Our work shows that monitoring attention processes can be efficiently done over a wide frontocentral area, including prefrontal electrodes [15,16]. In line with this, other research has shown that sampling from frontal electrodes might be sufficient to measure the migraine cycle [17]. As such, we found it relevant to use a simple and minimal setup with two electrodes for monitoring prefrontal activity.

Furthermore, we simplified the EEG analysis to adjust the extraction of relevant attention-related markers from a short sample, at the scale of 1 min, on the basis of template matching [18,19] of the marker, identified by the averaged ERP. It should be stressed that, while in the averaged ERP sample our marker is time-locked to the stimulus [15,16], we noted that the marker onset is much more flexible at the single trial level. Such large temporal variability (in the scale of many hundreds of milliseconds) is known in the literature, [19] and is larger for low frequency EEG activity. This has been related to the amplitude and phase of pre-stimulus oscillations [16]. Due to this large flexibility, of hundreds of milliseconds, in evoked response latency we did not time-lock the template matching at the single trial level. 

Based on this method, in this work we evaluated the applicability of following the dynamics of this simple marker during the migraine cycle. We hypothesize that, based on template matching, analyzing the attention-based markers from 1 min samples will distinguish between inter- and pre-ictal (12–48 h prior to the attack) electrophysiological patterns of cortical activity in migraine patients. The use of only the first minute of the sample aligns with the hypothesis cited above, in that the ERP differentiation and dynamics in migraine stems from changes in the response in the initial sample blocks. Such easy-to-extract attention-based markers have potential applications in the prediction of migraine attacks. Nevertheless, this is merely a pilot study and its results can only be viewed as preliminary.

## 2. Methods

### 2.1. Subjects

Thirteen migraine patients were recruited to participate in this study. Of these, 12 completed all 20 sessions and as such comprise the patient group; 1 patient left mid-study due to personal reasons and their data was excluded from the analysis. Patients were recruited through word of mouth and snowball sampling, starting with individuals who had contacted the headache clinic at Rambam Health Care Campus. The inclusion criteria were suffering from 2 to 10 attacks per month, and the commitment to participate in at least 20 sampling sessions within one calendar month. The exclusion criteria included any neurological or psychiatric disorders, presence of other chronic pain or hearing disorder and/or known eardrum injury. All patients underwent neurological evaluation by a migraine specialist for diagnosis verification. The patients maintained their regular preventive (if any) and acute treatment regimens during the sampling period. The study on migraine patients was performed in the Laboratory of Clinical Neurophysiology, Technion Faculty of Medicine and Rambam Health Care Campus. The data from migraine patients were compared with the samples of ten control subjects. The exclusion criteria of the control subjects included diagnosis of psychiatric or any neurological disorder, recent history of drug or alcohol abuse or dependence and hearing disorder and/or known ear drum impairment. These subjects were sampled twice a week for 8 weeks. Seven of the control subjects were tested in the Emotion–Cognition Research Center, Shalvata Mental Health Care Center, Hod-Hasharon, affiliated with the Sackler Faculty of Medicine, Tel-Aviv University. Three other subjects were tested in their homes after being evaluated by a physician and signing informed consent at either Shalvata Mental Health Center or Emek Medical Center.

The final study group consisted of 12 migraine patients (one patient did not complete the study and therefore was excluded from the analysis), 20–35 years; 11:1 F:M. The mean disease duration was 9.8 ± 6.3 years. The mean and maximal pain during a migraine attack were 6.0 ± 1.3 and 8 ± 1.3, respectively (VAS 0–10). The monthly attack frequency ranged from 2 to 10 (mean 4.7 ± 2.1). The number of migraine days per month varied from 2 to 15 days (mean 6.4 ± 3.5). Seven out of 12 patients had migraine with aura. Two patients had visual aura, 5 patients experienced signs of both visual and sensorimotor aura. During an attack, all patients had phonophobia, 11/12 had photophobia, 6/12 suffered from increased olfactory sensitivity, 2/12 suffered from increased taste sensitivity, 5/12 experienced vomiting and/or nausea. None of the patients utilized daily preventative treatment. Due to the small sample size we did not differentiate in the analysis between migraines with and without aura.

The study on migraine patients was approved by the local Helsinki Committee of Rambam Health Care Campus, approval number 0358-15-RMB. The study on control subjects was approved by the local Helsinki Committee of Shalvata mental health center, approval number 0010-15-SHA. All patients provided signed inform consent prior to participation in the study.

### 2.2. Experimental Procedure

EEG data were recorded from the NeuroSky MindWave single-channel system (NeuroSky Inc., San Jose, CA, USA—CE authorized), with one frontal electrode (~Fpz) and one reference electrode on the earlobe, at a sampling rate of 512 Hz. The MindWave EEG headset uses dry EEG electrodes. Sampled data were transferred through a wireless connection to the experiment computer for offline processing. Each sampling session involved 5 min of stimulus-free recording and 5 min of recording that was synchronized with an auditory oddball protocol. The oddball stimuli consisted of 1000 and 2000 Hz tones of 40 ms duration, presented binaurally at ~60 dB. The stimuli comprised a frequent tone (1000 Hz) presented 80% of the time, and a rare tone (2000 Hz) presented 20% of the time. The inter-stimulus interval was selected randomly within the range of 2 to 3 s. Before each sampling period, good skin contact was secured (the headset provides an index of contact level). Only data from the 1st minute of the stimulus-related samples were used in the analysis. The use of data from only the first minute was done in continuation of previous studies with other clinical populations [18,19]. It seems applicable specifically in studies of migraine due to the phenomenon of reduced electrophysiological habituation, which is well-established in the migraine literature [4]. It is believed that in migraine, early electrophysiological activity differentiates between various periods of the migraine cycle (e.g., inter-ictal vs. pre-ictal), and between migraineurs and control participants, but as the electrophysiological sample advances, this difference might average out [13]. The role of the oddball paradigm was to maintain a higher level of attention as is accepted in the literature. We did not differentiate between the 2 types of stimuli in data analysis.

All patients underwent 3–5 sampling sessions per week, with 24–72 h between subsequent sessions. At the beginning of each sampling session, the patient completed a questionnaire which addressed the following parameters: (a) Indication of whether a new attack took place since the previous sampling session, and if yes, an indication of the timing of the attack; (b) indication of whether pre-ictal (prodrome) and post-ictal (postdrome) symptoms were noted since the previous sampling session (the detailed list included mood changes, weakness, sleep or dietary changes, headache, etc.); (c) assessment of stress level on a visual analog scale (VAS), ranging from 0 (no stress) to 10 (severe stress). 

### 2.3. Electrophysiological Signal Analysis: Brain Engagement Index 

**Figure 1:**
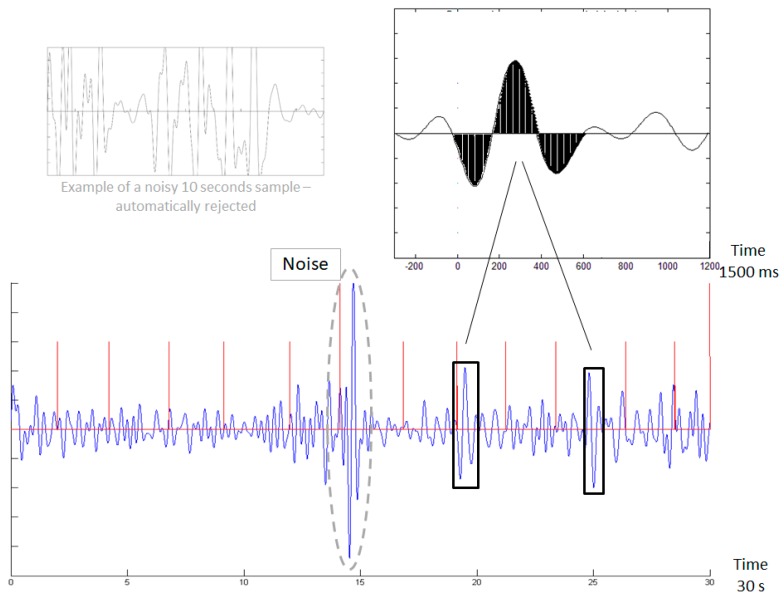
Demonstration of template matching.

The averaged ERP template [16] is emphasized in black in the top right inset of Figure 1. The new sample shown in the bottom of Figure 1 is scanned with a moving window, following normalization to [−1, 1] range. Whenever a match is found (black rectangles), it is counted. The Brain Engagement Index (BEI) is a normalization of this count to [0, 1] range. The sample in the bottom of Figure 1 shows an automatically rejected noisy sample (surrounded by a dashed gray line) as well. Stimulus times are marked with vertical lines. The red vertical lines denote the stimulus times. When the entire 10 s segment is noisy it is completely rejected, as shown in the top left grey inset of Figure 1, which has the same y-axis magnitudes as in the main graph, that is, −50 to +50 micro-volts. 

We computed the Brain Engagement Index for data from the 1st minute of the stimulus-related sample. The computation is based on template matching [18], a technique that uses a basic template, which is compared with the sampled signal. In this case the template was a 1500 milliseconds attention-related averaged ERP delta bandpass activity [16], which was matched with a moving window of the same size in the sampled signal. Matching was done in the following manner: (i) The one minute sample was divided into segments of 10 s. (ii) Each segment was filtered in the delta bandpass 1–4 Hz. (iii) Data points in the filtered segment were normalized into the [−1, +1] range, where −1 denotes the most negative deflection within the filtered segment, and +1 denotes the most positive deflection within the filtered segment. (iv) The process of filtering and normalization, to [−1, +1] range was also done for the 1500 averaged Delta ERP wave presented in the top inset of Figure 1 to generate the template (taken from [16]). (v) The normalized sampled segment was scanned by a moving window of 1500 ms with 1 sampling point steps. (vi) The averaged distance (absolute difference between normalized amplitudes) between the moving window data and the template and the template opposite was computed. (vii) If the averaged distance was less than the threshold value (0.5 for the template or template opposite, see Figure 1), a match count was made, provided no other match was found in a previous window partly-overlapping the current one. The 0.5 threshold was selected after meticulous manual evaluation of multiple samples in this study as well as in two other studies with different clinical populations [18,19] for the identification of sufficiently distinct patterns, which are not over-prevalent. (viii) If the averaged distance was more than the threshold, a no-matches count was taken, provided no other no-match count was found in a previous overlapping window. (ix) The BEI (Brain Engagement Index) is the division of the matches count to the no-matches count, which is again based on manual evaluation of multiple samples from this and other studies [18,19]. The BEI maximal value is set to +1, and therefore BEI is on a scale of 0–1. It should be noted that threshold selection, which might lead to possible false positive or false negative matches, would hopefully average out as the final index is based on tens of template matches over 1 min of sampling. (x) For every 1500 milliseconds window we also computed the ratio of the standard deviation to the mean of absolute activity (again sign insensitive, as was presented above regarding template matching, which is compared to both template and template opposite). Based on our manual inspection, if this ratio is greater than 1 then the sampling is likely to be noisy and these 1500 milliseconds samples were rejected and not included in the above computation. If multiple (more than 1) non-overlapping 1500 millisecond windows were rejected within a given 10 s segment, the entire segment was automatically rejected (for example, see the top left inset of Figure 1). At least 3 consecutive 10-s segments were required to be valid for the generation of a BEI for the entire sample (again, based on manual evaluation of multiple samples from this and other studies [18,19]). Otherwise, the entire sample was rejected as noisy and was not included in the next steps of analysis. If the samples of the first day or first two days were rejected as noisy, the subject was instructed to close his/her eyes in consequent samples. While this approach reduced overt noise, especially when prefrontal recording is considered, there is always concern regarding the impact of “milder” EMG/EOG noise sources, and especially in the delta bandpass in this region there is concern regarding the effect of blinking [20]. There seems to be a range of overlap in which it is uncertain whether activity originates from the brain or from muscle activity. However, interestingly blinking is well related to attention [21] and to the migraine cycle [22]. Furthermore, it seems the pattern of “attentive” well-deferred blinking in the delta bandpass is highly similar to our template pattern of hundreds of milliseconds [20]. Therefore, we did not see a practical need to differentiate between EEG activity and blinking, beyond the basic noise cleaning described above. 

Based on large single-trial variability [23], matching was not locked in time to the stimuli. Furthermore, no distinction was made between the two types of stimuli. As can be drawn from the algorithmic description above, we used template matching, which is sensitive to amplitude but not waveform. This is based on our previous work, which showed that after filtering to a sufficiently narrow bandpass (as in the case of the current work), it is possible to use amplitude-based analysis without significant loss of information [15,16]. 

### 2.4. Data Analysis

The sampling days of each patient were categorized retrospectively, according to the reported times of attacks, as follows: (a) Ictal samples during which the patient reported an ongoing attack; (b) pre-ictal samples that preceded an attack by up to 48 h (thus 2 pre-ictal days can precede each attack); (c) post-ictal samples that followed each attack by up to 48 h (thus 2 post-ictal days can succeed each attack; we distinguished between the 1st and 2nd post-ictal day); (d) inter-ictal samples, which do not match the ictal, pre-ictal, or post-ictal categories described above. If a sample was overlapping (e.g., was both within 2 days after one attack and within 2 days before another attack), it was excluded from analysis. Control subjects were sampled 14–16 times over a period of 8 weeks (1–2 times each week).

The focus of analysis was on the changes between the inter-ictal and the pre-ictal period. We also compared the ictal period and the inter-ictal and pre-ictal periods, as well as the post-ictal period to all other periods. Statistical significance for sample-based analysis was analyzed using repeated measures ANOVA corrected for multiple comparisons. For subject-based analysis we used paired *t*-test by subject for comparing the average BEI in the 4 periods (inter-ictal, pre-ictal, ictal and post-ictal). We further compared the 4 periods with the samples of the control subjects. Statistical significance was defined as *p* ≤ 0.05. In addition, we evaluated the changes of the post-ictal EEG dynamics by comparing the 1st and 2nd post-ictal days with a one-tailed, paired sample *t*-test.

Behavioral data from the inter-ictal and the pre-ictal periods were compared based on the onset of new pre-ictal symptoms and on measuring differences in VAS reports of stress. The onset of new pre-ictal symptoms on a specified sampling day was defined as such if symptoms were newly reported, and had not been reported during the previous sampling day. We focused on newly reported symptoms because this yielded some differentiation between the inter-ictal and pre-ictal periods. Such differentiation was not evident by simply comparing the ratios of days with reported symptoms, without consideration of whether the symptoms were also reported in the preceding days. To that aim, we calculated the ratio between the days with newly reported symptoms and the total number of sampling days (“report ratio”) in the inter-ictal period and in the pre-ictal period for each subject. We then compared the inter-ictal and pre-ictal report ratios across subjects and evaluated the significance of this comparison using a *t*-test. For the stress VAS evaluation, we used the absolute difference between the stress VAS report on each sampling day and the report on the previous day. We focused on absolute difference in stress VAS because it yielded some differentiation between the inter-ictal and the pre-ictal periods. Such differentiation was not evident by averaging the raw VAS scores or by averaging the non-absolute VAS differences. Therefore, we averaged the absolute VAS difference for the inter-ictal period and pre-ictal period for each subject. We then compared the inter-ictal and pre-ictal averaged differences across subjects and evaluated the significance of this comparison using *t*-test.

## 3. Results

### 3.1. Sample Counts

After noisy samples were automatically excluded, the analysis was based on 43 inter-ictal (3.58 ± 1.38 per subject), 19 pre-ictal (1.58 ± 1.24 per subject), 13 ictal (1.08 ± 1.24 per subject), and 22 post-ictal samples (1.83 ± 1.03 per subject, of which 10 samples were taken on the first post-ictal day and 12 samples on the second post-ictal day). For the control subjects, 99 non-noisy samples were included. The control subjects were comprised of two age groups: 5 subjects aged 23–32 (3:2 F:M), which were matching in age with the migraine subjects; and 5 subjects aged 59–75 (3:2 F:M).

### 3.2. Lack of Significant Evidence in the Behavioral Measures 

No significant difference was observed for the onset of newly detected symptoms between pre- and post-ictal migraine phase (*p* = 0.34). Similarly, no differences in stress VAS between pre- and post-ictal phases were found (*p* = 0.16). 

### 3.3. Brain Engagement Index Dynamics between Inter-Ictal and Pre-Ictal Periods (as well as the Ictal Period) and Comparison with the Post-Ictal Period and with Control Data

**Figure 2:**
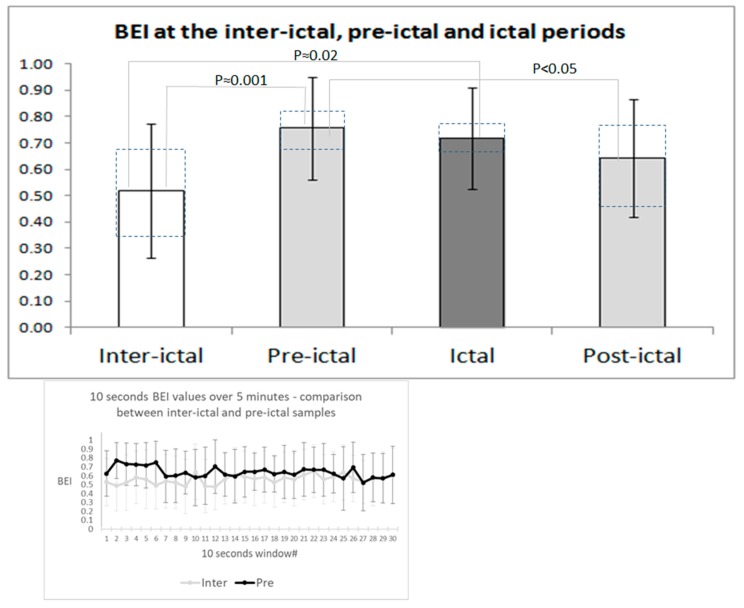
Brain Engagement Index (BEI) dynamics between migraine periods.

Lower BEI values were observed in the inter-ictal compared with the pre-ictal (*p* = 0.001, corrected for multiple comparisons) and ictal (*p* = 0.02, corrected for multiple comparisons) periods. The difference between the pre-ictal and ictal periods is not significant. The two post-ictal days did not differ significantly from the two pre-ictal samples (*p* = 0.09), ictal samples (*p* = 0.16) and inter-ictal samples (*p* = 0.08). Control samples differed significantly from the pre-ictal samples (*p* < 0.01), but not from the inter-ictal (*p* = 0.06), ictal (*p* = 0.09) and post-ictal samples (*p* = 0.51). In Figure 2, dashed squares present ranges between the first and third quartile in each condition. The bottom inset of Figure 2 shows the mean BEI (±standard deviation) for every 10 s segment of the entire 5 min sample for the major comparison between inter-ictal samples and pre-ictal samples. In accordance with reports in the literature, we observed higher starting values in the pre-ictal period, followed by significant habituation, which approach inter-ictal values.

Figure 2 presents the comparison of average Brain Engagement Indexes in the inter-ictal, pre-ictal, ictal, and post-ictal periods and for the control subjects. Data for the control subjects are summed for both age groups, as their BEI values were similar (0.62 ± 0.27 for the younger (age-matching) group, and 0.60 ± 0.25 for the older group, *p* = 0.35). Therefore, we used data for the entire control group to compare with the migraine samples.

The main focus of the study considered the possibility of identifying changes between the inter-ictal and pre-ictal and ictal periods. The difference between these periods was significant according to repeated measures ANOVA (F(2,72) = 8.57, *p* < 0.001). Post-hoc Tukey analysis indicated a significant difference in Brain Engagement Index values between the inter-ictal vs. pre-ictal periods (*p* = 0.001). The difference between the inter-ictal and ictal periods was also significant with Tukey HSD correction (*p* = 0.020). There was no significant difference between the pre-ictal and ictal periods (*p* = 0.27). The post-ictal period did not differ significantly from the pre-ictal (*p* = 0.09), inter-ictal (*p* = 0.08) and ictal (*p* = 0.16) periods. The control samples differed significantly from the pre-ictal samples (*p* < 0.01), but not from the inter-ictal (*p* = 0.06), ictal (*p* = 0.09) and post-ictal samples (*p* = 0.51).

In line with ANOVA results, the subjects-based paired *t*-test analysis of paired subject averages was significant between the inter-ictal and pre-ictal periods (*p* = 0.008), and between the inter-ictal and ictal periods (*p* = 0.003). There was no significant difference between the pre-ictal and ictal averages (*p* = 0.26). No significant differences were found between the paired post-ictal and inter-ictal samples, post-ictal and ictal samples, or pre-ictal and ictal samples (*p* = 0.06, *p* = 0.15 and *p* = 0.18, respectively). The bottom inset of Figure 2 shows the tendency for habituation of the BEI from high values in the first minute towards the inter-ictal level in the 5th minute. As presented above, this is in accordance with the literature regarding habituation in migraine, and seems to support the use of the first minute sample data throughout this study.

### 3.4. Brain Engagement Index Dynamics in the Post-Ictal Period

**Figure 3:**
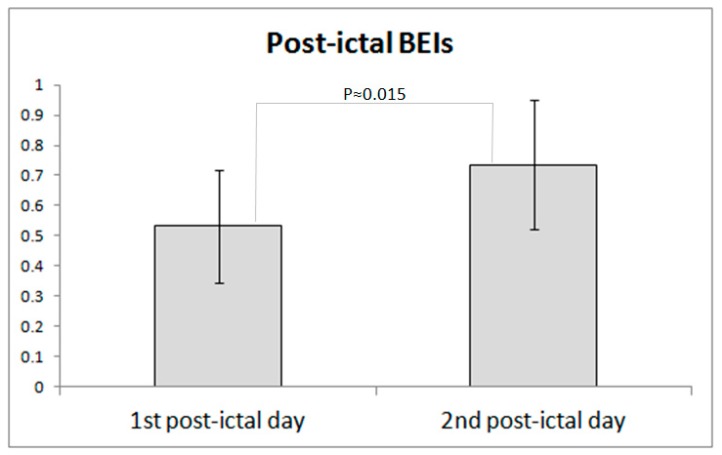
Comparison of the average BEI for the first and second post-ictal day. A higher BEI was observed in the second compared with the first post-ictal day (*p* = 0.015, paired *t*-test).

Figure 3 presents the comparison of the average Brain Engagement Index values for the first and second post-ictal day. The difference is statistically significant in a paired sample *t*-test (*p* = 0.015, one way).

### 3.5. Peri-Ictal Dynamics (before the Attack Prediction Section)

**Figure 4:**
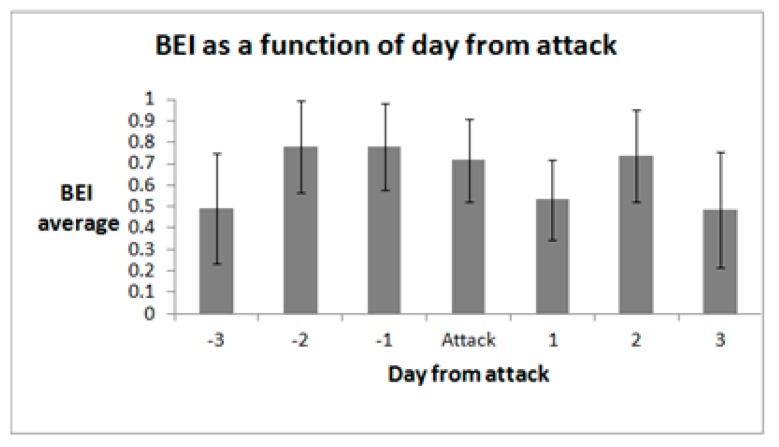
Dynamics of BEI by day from attack. Only non-overlapping samples were included for analysis (e.g., if a sample was taken up to two days after one attack and within two days of the next attack, it was excluded from analysis).

Figure 4 presents the daily dynamics before and after a migraine attack. Only non-overlapping samples were included (e.g., if a sample was taken up to two days after one attack and within two days before the next attack, it was excluded from analysis).

### 3.6. Attack Likelihood 

**Figure 5:**
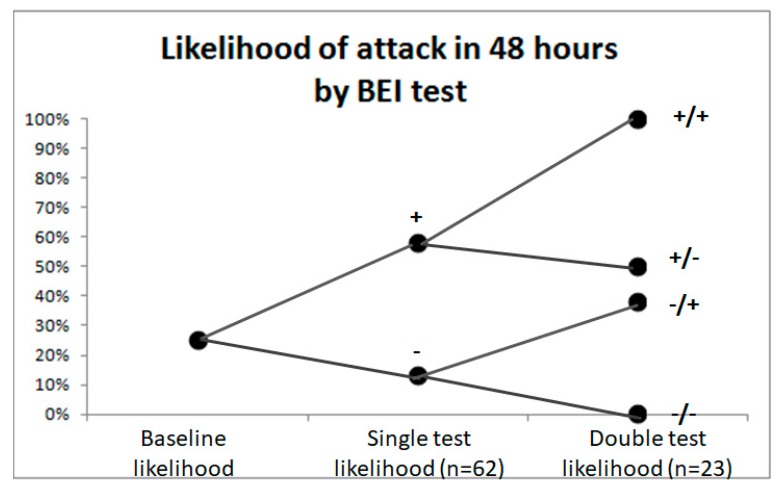
Likelihood of migraine attack in 48 h according to the BEI test.

The baseline likelihood of an attack in 48 h is ~25%, taking into account the frequency of attacks averaged over all patients. The result of a single BEI test improves attack prediction, and the results of two tests, taken one day after another, improves prediction further. A BEI value above threshold is denoted by “+”, and “–” denotes BEI values below threshold. The threshold is 0.5 for 10 out of the 12 patients. In 2 patients the average inter-ictal BEI was ~0.75 (with a pre-ictal increase towards 1). For these patients, a threshold of 0.75 was selected. Only tests from the inter-ictal and pre-ictal periods were included in the prediction analysis. Missing tests are due to noise or skipped sampling days. In the double (2 day) test shown in Figure 5, the left mark is for the first day and the right mark for the second day. 

We calculated the predicted accuracy for the pre-ictal and ictal periods. Figure 5 presents the likelihood of attack within 48 h by the Brain Engagement Index test. Baseline likelihood of attack in 48 h is ~25% taking into account average attack frequency. The results of a single Brain Engagement Index test improved attack prediction and the results of two tests taken on consecutive days improved attack prediction even further. Only samples from the inter- ictal and pre-ictal periods were included in the prediction analysis. Missing samples are due to noise or skipped sampling days. The threshold is 0.5 for 10 out of the 12 of patients, which represents the average inter-ictal BEI for this group. For two patients, the average inter-ictal Brain Engagement Index was ~0.75 (with a pre-ictal increase toward 1). In these patients, the threshold was selected as 0.75. In the double (two day) test, the left mark is for the first and the right mark for the second day.

## 4. Discussion

Consistent with previously reported work [3,4,5,6,7,9,10,11], we found that the different periods in the migraine cycle differ in their electrophysiological characteristics. Specifically, we found that in general the Brain Engagement Index increases in the pre-ictal period, remains high during the ictal period, decreases on the first post-ictal day, increases on the second post-ictal day, then remains relatively low during the inter-ictal period. 

We suggested in earlier studies that the Brain Engagement Index is related to attention and increases with increased attention [15,16,18,19]. Similar dynamics of EEG/ERP markers have been related to changes in attention during the migraine cycle [6,7,10,11]. Others showed similar dynamics exist in ERP markers, which may be more closely related to sensation and perception [4,5]. However, the spatiotemporal overlap in ERP manifestation of perception-related and attention-related processes appears to be significant in the first 200 milliseconds after stimulus. ERP in this period seems to be highly affected by both attention- and perception-related processes. We recently showed a significant effect of attention-related processes on very early ERP waves (within a few milliseconds after stimulus onset) in attention deficit hyperactivity disorder (ADHD), as compared to control subjects [14,15]. This effect may stem from very early neurophysiological responses upon sensation- and perception-related processes. The effect of attention, described above, on these waves, whether neurophysiological or technical, can in principle involve all of the different modalities. 

Reduced habituation of early ERP waves was demonstrated in migraine for multiple sensory modalities [4,5]. Some of the findings of reduced habituation in migraine were demonstrated in short-latency cortical evoked potential waves, and even with short-latency sub-cortical waves [24]. Within the paradigm of evoked potentials, there is no conclusive evidence that such short-latency waves are affected by attention [25]. Thus, it might be considered supportive of a reduced habituation mechanism. However, this lack of evidence may be the result of limitations in the evoked potentials method (which relies mainly on averaging that is affected by lack of habituation), because there is strong evidence for such an early effect of attention both for early cortical activity in primary and secondary sensory regions [26] and for sub-cortical activity [27,28,29]. There is significant evidence in a large number of studies, based on neuropsychological evaluations, of differences in inter-ictal attention in migraineurs [30], but hardly any conclusive neuropsychological evidence for differences that could be traced only to perception. Taken together, it can be hypothesized that all inter-ictal ERP differences may stem from variations in attention-related processes. However, it should be noted that this is merely a possibility. The current work alone cannot be viewed as a comprehensive proof that attention changes underlie the electrophysiological dynamics reported during the migraine cycle in this study and by others.

Previous works have reported pseudo-normalization of ERP-based electrophysiological markers in the transition from the inter-ictal to the pre-ictal period [10,11]. We obtained similar findings, with a matching increase in the Brain Engagement Index, even more profound when compared to the BEI in healthy controls. Such an increase may in fact stem from the focus of our analysis to the first sampling minute, without the “normalizing” effect of relative habituation, described above. It is important to note that there is also an increase in the Brain Engagement Index from the first to the second post-ictal day. Assuming that these findings relate to changes in attention level, our new observation concerning the post-ictal dynamics may represent true normalization and even over-sensitization, which is later reduced during the inter-ictal period. This suggests that whenever increased Brain Engagement Index values are encountered in the inter-ictal period, they are caused by either (a) “pseudo-normalization,” which indicates a pending attack that was eventually avoided, or (b) true but temporary normalization of the attention abilities, presumably similar to the second post-ictal day. 

In a detailed theoretical work [31], we suggested that ERP changes reported in the literature during the migraine cycle may originate from changes in attention level. According to this model, the apparent pseudo-normalization, also reported by others [6,7,10,11], might represent enhanced attentive response to auditory stimuli due to global reduction in relevant brain activity and greater sensitization (see detailed explanation in [28]). We further suggested that these changes in attention level are the result of the effect of stress. Stress level may affect the migraine cycle [32] and its effect on electrophysiological markers of attention are well established. It is generally accepted that stress reduces attention response to the majority of non-stressful stimuli, while the attention response to the stressing stimuli increases [33]. 

Our behavioral results demonstrated only a modest and non-significant change in self-reported stress in the transition from the inter-ictal to the pre-ictal period. This, however, may be the result of limited sensitivity when measuring stress on a VAS scale. We did not observe a clear tendency for increased stress in the inter-ictal—pre-ictal transition, and a decrease in stress was reported as frequently as an increase. Others have observed an occasional decrease in stress in the inter-ictal—pre-ictal transition. This phenomenon, termed “stress letdown” [34], is consistent with our theoretical model. It is beyond the scope of the current paper to discuss this in detail. Nevertheless, we suggested elsewhere the role for sub-cortical networks in this phenomenon as well as in the overall dynamics of the migraine cycle [31]. 

## 5. Conclusions

Irrespective of the correctness of this or any other theory, the present work demonstrates the preliminary feasibility of migraine attack prediction with an easy-to-use electrophysiological tool. Such a tool may also make it possible to evaluate preventive interventions, which often consist of non-medical stress-reducing techniques. Nevertheless, it should be stressed that the current study is based on small groups of patients and controls and can only be viewed as a pilot which provides further supporting evidence regarding the potential feasibility of an easy-to-use tool for attention measuring, which is additive to previously published studies [18,19] with other clinical populations. It will be necessary to find a way to reduce the number of samples marked as noisy, for example, by methods of artifact correction instead of excluding multiple samples, before such a tool might become relevant in practice. Furthermore, it is necessary to first reassess the results of this pilot study using a large prospective design. Note that our results suggest that the BEI only had predictive power before an attack and not during the post-ictal period, and is especially problematic on the second post-ictal day. For the post-ictal period, prediction should probably be based on a return to normal functionality. It also seems to be imperative to evaluate the association between the BEI dynamics and the self-reported patient attention or concentration dynamics. Nevertheless, this work suggests that it is especially valuable to further investigate the utilization of BEI for attack prediction, as it is extremely simple to extract this information.
